# Circ_0001367 inhibits glioma proliferation, migration and invasion by sponging miR-431 and thus regulating NRXN3

**DOI:** 10.1038/s41419-021-03834-1

**Published:** 2021-05-25

**Authors:** Liang Liu, Peng Zhang, Xuchen Dong, Haoran Li, Suwen Li, Shan Cheng, Jiaqi Yuan, Xuejun Yang, Zhiyuan Qian, Jun Dong

**Affiliations:** 1 Department of Neurosurgery, The Second Affiliated Hospital of Soochow University, 215004 Suzhou, China; 2 Rugao Hospital Affiliated to Nantong University, 226500 Nantong, Jiangsu China; 3 Medical College of Soochow University, 215123 Suzhou, Jiangsu China; 4 Department of Neurosurgery, Tianjin Medical University General Hospital, 154 Anshan Road, 300052 Tianjin, China

**Keywords:** CNS cancer, Long non-coding RNAs

## Abstract

Many studies have reported that circular RNAs play a vital role in the malignant progression of human cancers. However, the role and underlying mechanism of circRNAs in the development of gliomas have not been fully clarified. In this study, we found that circ_0001367 was downregulated in glioma tissues and showed a close correlation with glioma patient survival. Functional assays demonstrated that upregulation of circ_0001367 could suppress the proliferation, migration and invasion of glioma cells in vitro and inhibit glioma growth in vivo. Furthermore, bioinformatics analysis, luciferase reporter assay and RNA immunoprecipitation assay indicated that circ_0001367 can serve as a sponge for miR-431 and that miR-431 acts as an oncogene by regulating neurexin 3 (NRXN3). In addition, rescue experiments verified that circ_0001367 could regulate both the expression and function of NRXN3 in a miR-431-dependent manner. In conclusion, circ_0001367 functions as an suppressor in glioma by targeting the miR-431/NRXN3 axis and may be a promising therapeutic target against gliomas.

## Introduction

Gliomas are the most common primary intracranial malignant tumours, and their treatment represents a significant challenge for neuro-oncologists^1,2^. The therapeutic outcome of high-grade gliomas is very poor, and the 5-year survival rate of glioblastoma multiforme is <10%^3^. Advancements in in-depth genomics, epigenetics and tumour immunology research have promoted some progress in the molecular diagnosis and development of new therapeutics for glioma. However, there is still an unmet clinical need for improving glioma treatment efficacy and patient outcomes^4^. The development of novel effective treatments highly relies on elucidating the fundamental molecular mechanisms of glioma development.

Circular RNA (circRNA) is a type of non-coding RNA (ncRNA) with a closed-loop structure, without a 5′ cap and a 3′ poly(A) tail, mainly located in the cytoplasm or stored in exosomes^5,6^. CircRNAs are not affected by RNA exonucleases, and their expression is more stable and not easy to degrade^7^. Most of circRNAs are made by exon circularization, and some of them are produced through intron circularization^8^. Twenty years ago, circRNAs were first identified from yeast mitochondria and hepatitis B virus as by-products of abnormal shearing with no regulatory function^9^. In 2013, it was shown that circRNAs function as sponges of miRNAs, providing a new area for circRNA research^10^. With the rapid development of RNA sequencing technology and bioinformatics analysis, tens of thousands of circRNAs have been identified in human tissue transcriptomics. Currently, circRNAs have been proven to widely exist in eukaryotes and have the characteristics of tissue specificity and disease specificity. Many studies have shown that circRNAs are linked to biological growth and development, stress response and disease occurrence and development^11–13^. Recent studies have reported specific roles and functions of circRNAs in gliomas; however, their exact roles remain largely unknown and deserve further investigation.

MicroRNAs (miRNAs), a class of endogenous ncRNAs in eukaryotes, are approximately 20–25 nucleotides in length and have regulatory functions in various physiological and pathological processes^14^. Mature miRNAs are produced by a series of long transcripts through a series of nuclease cleavages and assemble into an RNA-induced silencing complex^15^. According to the degree of complementarity, different miRNAs guide the silencing complexes to degrade target mRNAs or repress mRNA translation. MiRNAs have been reported to act as circRNA targets to participate in tumour initiation and progression in recent years^16,17^. However, the role of the circRNA–miRNA regulatory network in gliomas has not been well studied.

In the present study, we aimed to explore the differences in the expression patterns of circRNAs between malignant gliomas and their corresponding adjacent normal brain tissues (NBTs). Circ_0001367 was identified as a significantly differentially expressed circRNA. Integrated analysis of both clinical data and gain- and loss-of-function assays was performed to clarify whether circ_0001367 was significantly correlated with the prognosis of glioma patients. Based on luciferase reporter assays, RNA immunoprecipitation (RIP) assays and functional assays, the sponging and regulatory activity of circ_0001367 were investigated to elucidate its modulatory roles in glioma progression.

## Materials and methods

### Clinical specimens

Clinical glioma tissue specimens (*n* = 50) and adjacent NBTs were collected from patients who underwent surgical removal and were diagnosed with glioma according to World Health Organization (WHO) pathological criteria at the Department of Neurosurgery, Second Affiliated Hospital of Soochow University. The pathological diagnosis of glioma was independently achieved by two senior and experienced pathologists. Ethical approval was obtained from the Second Affiliated Hospital of Soochow University. Informed consent was obtained from all subjects.

### Cell culture and transfection

Normal human astrocytes (NHAs) were obtained from JENNIO Biological Technology (Guangzhou, China). Glioma cell lines (A172, LN229, T98G, U118, and U138) were obtained from American Type Culture Collection (ATCC). All the cell lines were authenticated by short tandem repeat and were cultured in Dulbecco’s modified Eagle’s medium (Gibco, NY, USA) supplemented with 10% foetal bovine serum (ScienCell, LA, USA) and maintained in an incubator containing 5% CO_2_ at 37 °C.

The short hairpin RNAs (shRNAs), miR-431 mimics, miR-431 inhibitors, overexpression plasmids, and the corresponding negative controls used in this study were all purchased from GenePharma (Shanghai, China) and transfected into cells using Lipofectamine 3000 (Invitrogen, Carlsbad, CA, USA) according to the manufacturer’s instructions. Oligonucleotide sequences applied for cell transfection are listed in Table S1.

### RNA extraction and quantitative reverse transcription–polymerase reaction (qRT-PCR)

Total RNA was extracted from tissues and cells using TRIzol (Invitrogen) as previously described^18^. qRT-PCR was conducted to measure the abundance of transcripts using SYBR PremixEx Taq (Vazyme, China) with the 2^−ΔΔCt^ method. qRT-PCR was performed on each sample in triplicate. The primers used in this study are listed in Table S2.

### RNase R treatment

Total RNA was incubated with or without RNase R (3 U/mg, Epicenter, WI, USA) for 30 min at 37 °C. Next, RNase R-treated RNA was purified with the RNeasy MinElute Cleaning Kit (Qiagen, Dusseldorf, Germany). Then qRT-PCR was conducted to measure the expression of circ_0001367 and the linear transcript Kelch-like family member 24 (KLHL24).

### Luciferase reporter assay

The wild-type and mutant fragments in the 3′-untranslated region of circ_0001367 (circ_0001367-MUT, CAG UUG UGU UCC GUA UAG GUG GG) and neurexin 3 (NRXN3) (NRXN3-MUT, AGC UCC GUA AAG CUA GAA CGA AA) related to the miR-431-binding site were synthesized and inserted into pMIR-REPORT vectors. Next, pMIR-REPORT vectors, together with miR-431 mimics or the corresponding negative control, were transfected into glioma cell lines. Precisely 48 h later, a dual-luciferase reporter assay system (Promega, WI, USA) was used to detect the luciferase activity of tumour cells.

### RNA immunoprecipitation

The RIP assay was performed using the RIP Kit (Millipore, Billerica, MA, USA), according to the manufacturer’s instructions. Cells were washed in phosphate-buffered saline and lysed thoroughly in lysis buffer, followed by centrifugation at 12,000 × *g* for 30 min, and the supernatant was harvested. Next, the supernatant was incubated with magnetic beads coupled with anti-Argonaute-2 (AGO2, Abcam, Cambridge, UK) or anti-IgG (Abcam) antibody for 6 h at 4 °C. After RIP, reverse transcription was performed. Finally, the expression of circ_0001367 was analysed using qRT-PCR.

### Fluorescence in situ hybridization (FISH)

The probes and FISH Kit used in this assay were purchased from GenePharma. First, tissues were fixed in 4% paraformaldehyde, dehydrated in ethanol, embedded in paraffin and sliced into sections. Next, the sections were treated sequentially with dimethylbenzene xylene (15 min), anhydrous ethanol (5 min), alcohol (10 min) and proteinase K (30 min). Then the sections were hybridized at room temperature in hybrid solution (1 μl) containing hsa_circ_0001367 or miR-431 probes overnight. After washing, cell nuclei were stained with DAPI (4′,6-diamidino-2-phenylindole; Invitrogen) at room temperature for 5 min. Finally, images were captured with a fluorescence microscope (Olympus, Tokyo, Japan).

### Western blot analysis

Total protein from the tissue and cells was extracted using RIPA buffer (KenGEN, Shanghai, China). Protein concentration was determined with the BCA Protein Assay Kit (Beyotime, Shanghai, China). The following steps were described previously^19^. The relevant antibodies used are listed in Table S3.

### Colony formation assay

Cells (3 × 10^3^) were seeded into cell culture dishes (35 mm, Corning, USA) and cultured for 14 days in an incubator containing 5% CO_2_ at 37 °C. Then the cells were fixed in 4% paraformaldehyde for 10 min, followed by crystal violet staining for 20 min. Transparent films of the grid were used to count the number of clones.

### 5-Ethynyl-20-deoxyuridine (EdU) assay

EdU assay was performed as described previously^20^. Briefly, cells (2.0 × 10^4^) were seeded into 96-well plates and cultured in an incubator containing 5% CO_2_ at 37 °C overnight. After incubation with EdU (RiboBio, Guangzhou, China) for 2 h, cells were fixed with 4% paraformaldehyde and stained sequentially with Apollo dye solution (RiboBio) and DAPI (Invitrogen), and images were captured using a fluorescence microscope (Olympus).

### Transwell assay

Transwell assays were performed to evaluate the migration and invasion abilities of glioma cells. The difference between invasion and migration was the upper chambers coated with or without Matrigel (50 µl, 1:8 dilution, BD, NJ, USA). The protocol was carried out as previously described^21^.

### Immunohistochemistry (IHC)

Tissue section preparation was the same as the FISH assay. IHC was performed as previously described^19^. Briefly, the tissue sections were incubated with the primary antibody at 4 °C overnight, then the biotinylated secondary antibody at room temperature for 2 h, followed by ABC-peroxidase for 1 h. Next, diaminobenzidine and haematoxylin were applied to stain and counterstain the tissue section. Finally, images were captured under a microscope (Olympus) and evaluated in a blinded manner by two senior pathologists independently.

### Terminal deoxynucleotidyl transfer-mediated dUTP nick end labelling staining (TUNEL)

The TUNEL assay in this study was conducted with a TUNEL Assay Kit (Beyotime) and Cell Death Detection Kit (Roche, Basel, Germany) according to the manufacturer’s instructions. The tissue section preparation was the same as the FISH assay. After being fixed and rinsed, tissue sections were stained using the fluorescein isothiocyanate-end labelling method. A Cell Death Detection Kit was then applied to detect apoptotic cells. The images were captured with a fluorescence microscope (Olympus).

### Intracranial tumour mouse model

LN229 cells (2 × 10^6^) stably expressing luciferase and transfected with sh-circ_0001367, sh-NRXN3 or the corresponding negative controls were injected into the right caudate nucleus of female Balb/c nude mice (4–6 weeks old, 10 mice per group), which were purchased from the Beijing Laboratory Animal Center (Beijing, China) and allocated randomly. A bioluminescence imaging system (IVIS Lumina II, Caliper, USA) was applied to monitor tumour growth on the indicated days. The Living Images software package (Caliper Life Science, Waltham, MA, USA) was used to determine the integrated flux of photons (photons/s). The xenograft tumours were harvested for western blot analysis, IHC and TUNEL assay. Animal experiments were approved by the Institutional Animal Care and Use Committee of Second Affiliated Hospital of Soochow University. No blinding was done in this experiment.

### Statistical analysis

The data in this study were analysed with the GraphPad Prism software (Version 8.0.2, (CA, USA). The results are presented as mean ± SD. One-way analysis of variance was used to evaluate the differences among at least three groups. Student’s *t* test was used to determine the differences between groups. A *P* value <0.05 was accepted as statistically significant. All experiments were repeated three times independently.

## Results

### Circ_0001367 was downregulated in glioma and associated with poor prognosis

**Fig. 1:**
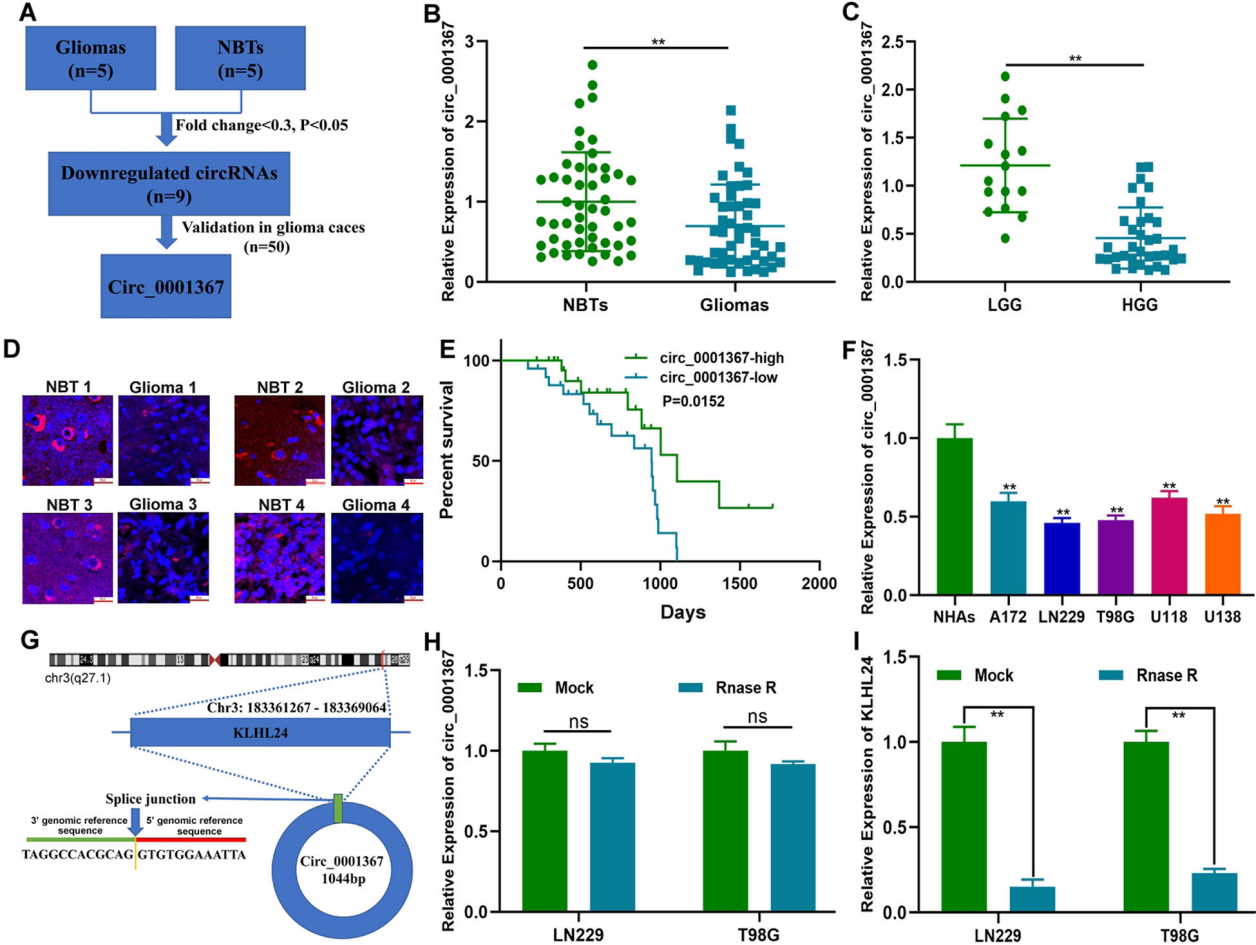
Circ_0001367 was downregulated in glioma and associated with a poor prognosis. **A** Schematic illustration of the identification of circRNAs downregulated in glioma tissues compared with adjacent NBTs. **B** The expression of circ_0001367 was detected using qRT-PCR in glioma surgical specimens and paired adjacent NBTs. **C** Expression of circ_0001367 in surgical specimens of low-grade glioma (LGG) and high-grade glioma (HGG). **D** The expression of circ_0001367 was detected using FISH in glioma specimens and paired adjacent NBTs. (Red: circ_0001367, Blue: DAPI). **E** Kaplan–Meier analysis of glioma patients with high or low expression of circ_0001367. **F** qRT-PCR was performed to verify circ_0001367 expression in glioma cell lines. **G** Schematic illustrations showing the genomic loci of circ_0001367. **H**, **I** RNase R treatment indicated that circ_0001367 RNA was resistant to RNase R in LN229 and T98G cells. Each experiment was performed in triplicate. Data are expressed as mean ± SD, **P < 0.01.

To explore the role of circRNAs in glioma development and malignant progression, circRNA expression profiles in five surgical specimens of glioma patients and the corresponding adjacent NBTs were analysed. According to the profiles, nine circRNAs were downregulated significantly (fold change <0.3 and *P* < 0.05) in glioma tissues (Fig. 1A and Table S4). The expression patterns of the 9 circRNAs in 50 cases of gliomas and paired adjacent NBTs were validated by qRT-PCR. Circ_0001367, which has never been reported to be involved in glioma progression, had significantly lower expression levels in gliomas than in adjacent NBTs (Fig. 1B, C). Furthermore, circ_0001367 expression in glioma parenchyma and adjacent NBTs was also tested by FISH, which showed that circ_0001367 was downregulated in gliomas (Fig. 1D). Kaplan–Meier analysis indicated that glioma patients with low circ_0001367 expression had a poor outcome (Fig. 1E). The expression of circ_0001367 in glioma cell lines was also detected. Similar to the data from clinical specimens, compared with NHAs, circ_0001367 was downregulated in glioma cell lines, especially in LN229 and T98G cells (Fig. 1F). According to circbase (http://www.circbase.org/), circ_0001367 was derived from KLHL24, and the splice junction is shown in Fig. 1G. Otherwise, compared with the linear transcript, the RNase R enzyme had no effect on circ_0001367, which solidified the circRNA characteristics of circ_0001367 (Figs. 1H, I and S1). Overall, these results documented that circ_0001367 was a stable circRNA closely related to glioma progression.

### Circ_0001367 inhibited the proliferation, migration and invasion abilities of glioma cells in vitro

**Fig. 2:**
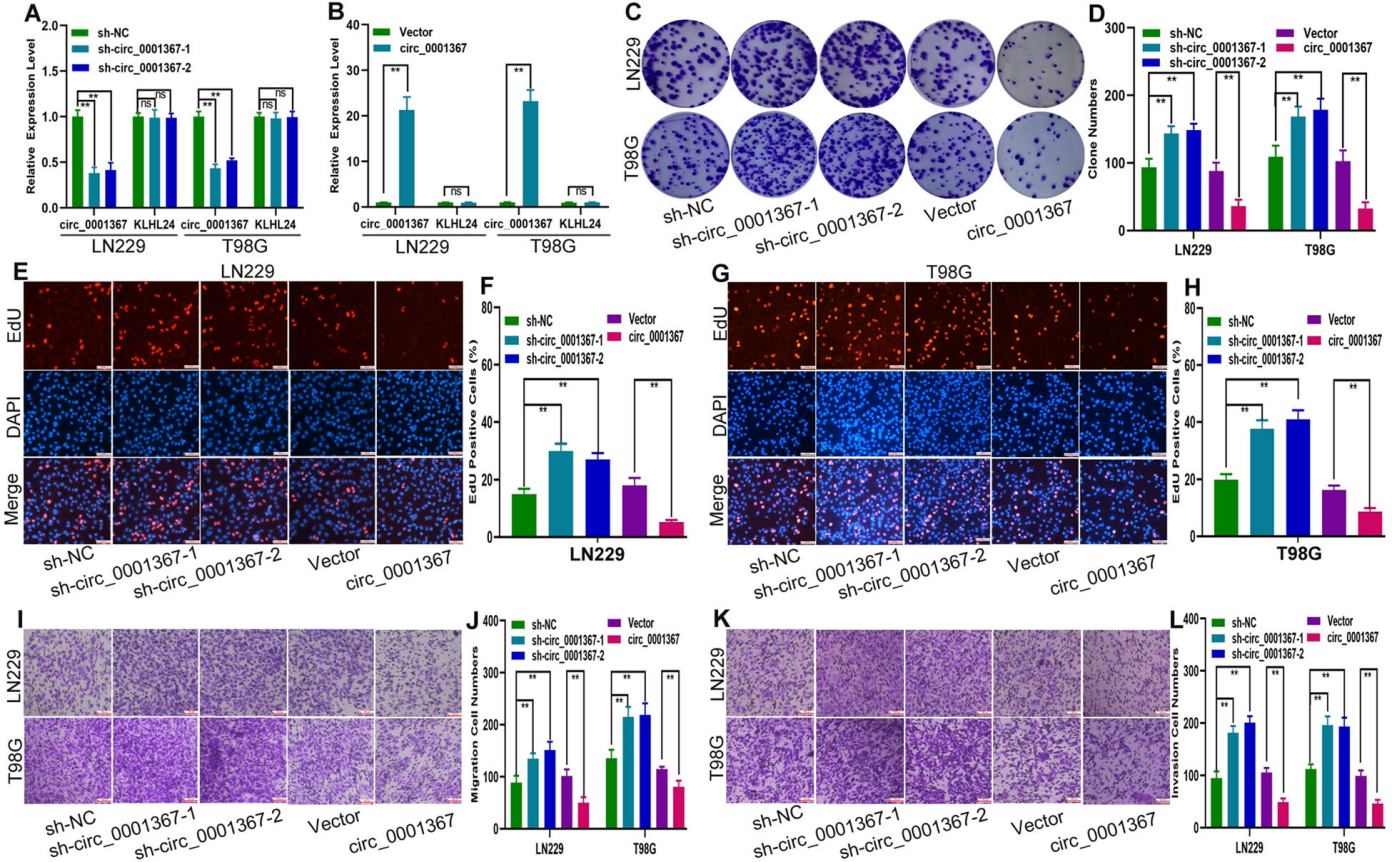
Circ_0001367 inhibited the proliferation, migration and invasion abilities of glioma cells in vitro. **A**, **B** qRT-PCR analysis of circ_0001367 expression in LN229 and T98G cells after transfection of the expression vector or shRNA. **C**, **D** Clone formation assays showed the clone formation ability of LN229 and T98G cells transfected with the expression vector or shRNA. **E**–**H** Cell proliferation analysis by EdU assay after LN229 and T98G cells were transfected with the expression vector or shRNA. **I**, **J** Cell migration analysis with a Transwell assay after glioma cells were transfected with the expression vector or shRNA. **K**, **L** Cell invasion was evaluated via Transwell assay after glioma cells were transfected with the expression vector or shRNA. Each experiment was performed in triplicate. Data are expressed as mean ± SD, **P < 0.01.

To evaluate the function of circ_0001367 in glioma cells, LN229 and T98G cells were transfected with short hairpin RNAs (shRNAs) targeting circ_0001367 (including sh-circ_0001367-1 and sh-circ_0001367-2), circ_0001367 overexpression plasmid and the corresponding negative control (Fig. 2A, B). Clone formation assays and EdU assays showed that silencing circ_0001367 expression promoted the proliferation of LN229 and T98G cells, whereas overexpressing circ_0001367 suppressed glioma cell proliferation (Fig. 2C–H). In addition, the Transwell assay indicated that silencing circ_0001367 expression promoted both the migration and invasion of LN229 and T98G cells. Overexpression of circ_0001367 decreased the migration and invasion abilities of these glioma cells (Fig. 2I–L).

### Silencing circ_0001367 promoted glioma growth in vivo

**Fig. 3:**
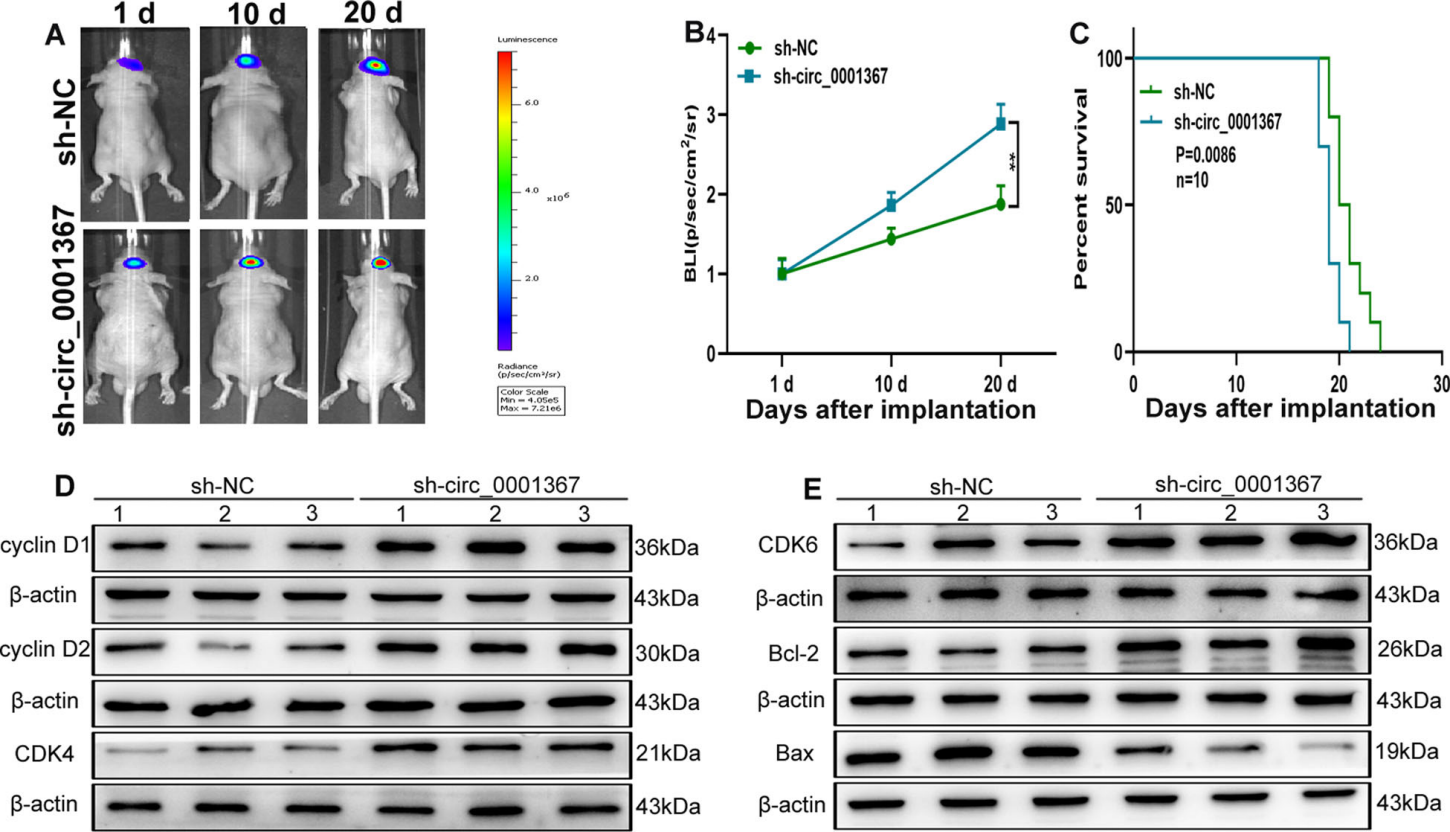
Silencing circ_0001367 promotes glioma growth in vivo. **A**, **B** Bioluminescent images of the intracerebral tumours of nude mice (10 mice in per group) on days 1, 10, and 20 after stereotaxic orthotopic implantation of glioma cells. **C** Overall survival was compared between the sh-circ_0001367 and sh-NC groups by Kaplan–Meier survival analysis. **D**, **E** The expression of proliferation- and apoptosis-related proteins was detected by western blot analysis. Data are expressed as mean ± SD, **P < 0.01.

To evaluate the effect of circ_0001367 on glioma growth in vivo, an intracranial xenograft model was established. Intracerebral injection of LN229 cells transfected with sh-circ_0001367 or the corresponding negative controls was performed with stereotaxic techniques. The results showed that silencing circ_0001367 promoted glioma growth in vivo (Fig. 3A, B), leading to shorter overall survival time in tumour-bearing mice (Fig. 3C). In addition, the expression of proliferation- and apoptosis-related proteins was detected by western blotting and IHC. Compared with those in the control group, cyclin D1, cyclin D2, cyclin-dependent kinase 4 (CDK4), CDK6, Bcl-2 and Ki-67 were upregulated, whereas Bax was downregulated in the sh-circ_0001367 group (Figs. 3D, E and S2A). The TUNEL assay indicated that TUNEL-positive cells decreased in the sh-circ_0001367 group as well (Fig. S2B).

### Circ_0001367 acted as a sponge for miR-431 in glioma cells

**Fig. 4:**
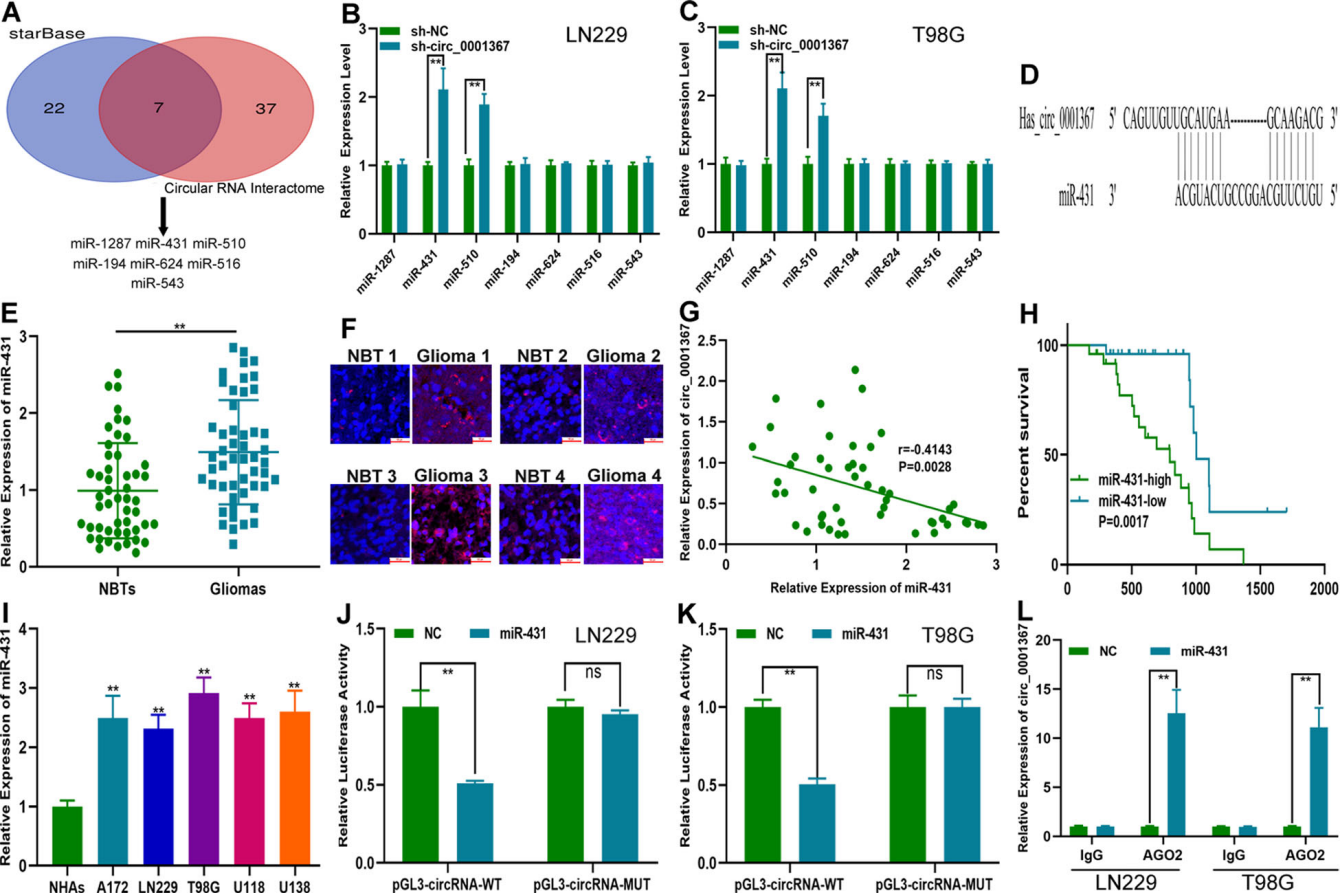
Circ_0001367 acted as a sponge for miR-431 in glioma cells. **A** A Venn diagram indicated that seven miRNAs might be biological targets of circ_0001367. **B**, **C** Relative expression of candidate miRNAs in LN229 and T98G cells transfected with sh-circ_0001367 or shNC. **D** Putative binding sites of miR-431 on circ_0001367. **E**, **F** The expression of miR-431 in glioma surgical specimens and matching adjacent NBTs was examined with qRT-PCR and FISH (Red: circ_0001367, Blue: DAPI). **G** Pearson correlation analysis was performed to determine the relationship between circ_0001367 and miR-431. **H** Kaplan–Meier survival analysis showed that the overall survival of glioma patients differed with high or low miR-431 expression. **I** MiR-431 expression in glioma cell lines examined using qRT-PCR. **J**, **K** Dual-luciferase reporter assays were performed to validate the association between circ_0001367 and miR-431. **L** RIP assay validated the association between circ_0001367 and miR-431. Each experiment was performed in triplicate. Data are expressed as mean ± SD, **P < 0.01.

Four mechanisms have been reported on the roles of circRNAs in tumour progression, among which competing endogenous RNA (ceRNA) is an important one^22,23^. Bioinformatics assays on the miRNA targets of circ_0001367 were carried out by analysing two online databases (circInteractome and starBase), and seven miRNAs were selected for further validation (Fig. 4A). The expression of seven miRNAs in LN229 and T98G cells transfected with sh-circ_0001367, circ_0001367 overexpression plasmid or the corresponding negative control was verified by qRT-PCR. The results showed that miR-431 and miR-510 had a close relationship with circ_0001367 (Figs. 4B, C and S3A, B). The bioinformatics assay from starBase indicated the exact sites at which circ_0001367 can bind to miR-431 and miR-510 (Figs. 4D and S3C). The expression pattern of miR-431 and miR-510 in clinical glioma specimens was investigated using qRT-PCR, which showed that miR-431 expression was upregulated in glioma tissues compared to the corresponding adjacent NBTs (Fig. 4E), whereas there was no significant difference in the expression of miR-510 between glioma tissues and their corresponding adjacent NBTs (Fig. S3D). FISH assays showed high expression of miR-431 in glioma tissues as well (Fig. 4F). Pearson correlation analysis indicated a significant correlation between the circ_0001367 level and miR-431 expression (Fig. 4G), whereas there was no correlation between circ_0001367 and miR-510 (Fig. S3E). Therefore, miR-431 was selected for further investigation. Kaplan–Meier analysis indicated that glioma patients with high miR-431 expression had a poor outcome (Fig. 4H). MiR-431 was upregulated in glioma cell lines as well (Fig. 4I). A luciferase reporter assay showed that miR-431 decreased the luciferase activity of circ_0001367-WT but not circ-0001367-MUT (Fig. 4J, K). Additionally, the RIP assay indicated that, compared with that in the input group, circ_0001367 expression was high in the AGO2 group (Fig. 4L). These results suggested that circ_0001367 can act as a sponge for miR-431 in glioma cells.

### MiR-431 directly bound NRXN3 to regulate the level of NRXN3

**Fig. 5:**
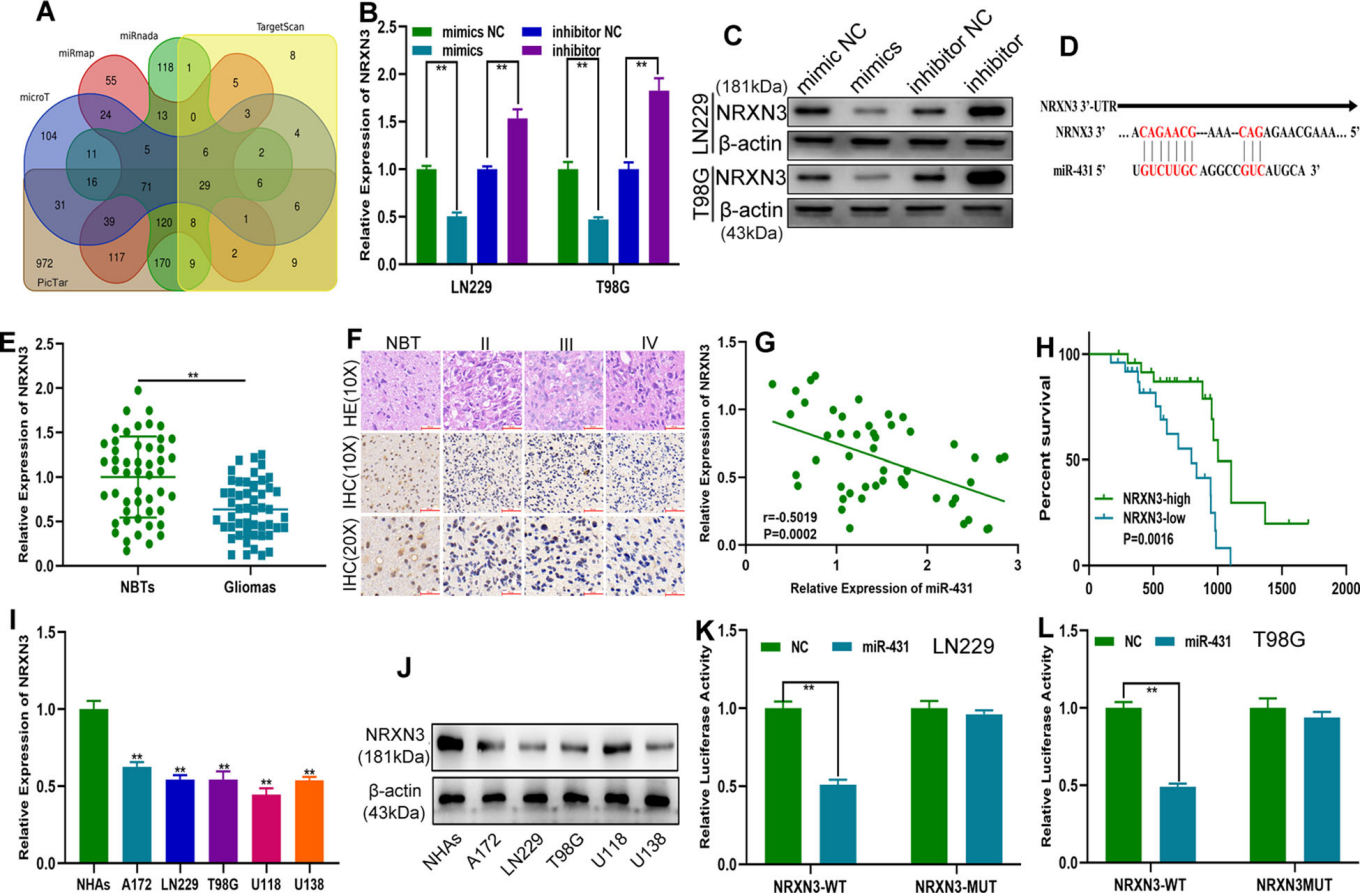
MiR-431 directly binds NRXN3 to regulate the level of NRXN3. **A** A Venn diagram indicating that 29 mRNAs may be biological targets of miR-431. **B**, **C** qRT-PCR and western blot analysis showing NRXN3 expression in glioma cells transfected with miR-431 mimics, miR-431 inhibitor, and the corresponding negative control. **D** The putative binding sites of miR-431 on NRXN3. **E** The expression of NRXN3 in clinical glioma specimens and matching adjacent normal brain tissues (NBTs) was examined using qRT-PCR. **F** The expression level of NRXN3 in different malignancy grades of glioma detected using immunohistochemistry. **G** Pearson correlation analysis was performed to determine the relationship between NRXN3 and miR-431. **H** Kaplan–Meier survival analysis showed differences in the overall survival of glioma patients with high or low NRXN3 expression. **I** NRXN3 expression in glioma cells examined by qRT-PCR. **J** NRXN3 expression in glioma cell lines was examined using western blot analysis. **K**, **L** Dual-luciferase reporter assay to validate the association between NRXN3 and miR-431. Each experiment was performed in triplicate. Data are expressed as mean ± SD, **P < 0.01.

To reveal the role of circ_0001367 in glioma progression and identify the target gene of miR-431, five online databases (PicTar, TargetScan, miRmap, miRnada and microT) were explored. Through bioinformatics analysis, 29 genes were screened for further validation (Fig. 5A and Table S5). The expression level of these candidate genes in a public database (GEPIA) was searched, and only two of them (serine rich and transmembrane domain containing 1 (SERTM1), and NRXN3) were downregulated in gliomas (Fig. S4). SERTM1 is located in chromosome 13, and its role in human physiology and pathology has not been extensively studied yet. NRXN3 is a presynaptic adhesion molecule, which belongs to the neurexin genes (NRXN1, NRXN2, and NRXN3), and mainly regulates neurotransmitter release^24^. Then, LN229 and T98G cells were transfected with miR-431 mimics, miR-431 inhibitor, or corresponding negative controls. qRT-PCR and western blot analysis indicated that only NRXN3 showed a close relationship with miR-431 (Figs. 5B, C and S5). The binding site between NRXN3 and miR-431 was shown in Fig. 5D. The expression pattern of NRXN3 in clinical glioma specimens was investigated, which showed that NRXN3 was downregulated in glioma tissue (Fig. 5E) and associated with WHO malignancy grades of gliomas (Fig. 5F). Pearson correlation analysis indicated a significant correlation between NRXN3 and miR-431 expression (Fig. 5G). In addition, Kaplan–Meier analysis indicated that lower NRXN3 indicated a poor outcome (Fig. 5H). The expression level of NRXN3 in glioma cell lines was consistent with that in clinical specimens (Fig. 5I, J). Finally, a luciferase reporter assay showed that miR-431 decreased the luciferase activity of NXRN3-WT but not NXRN3-MUT (Fig. 5K, L), suggesting that NRXN3 was the direct target of miR-431. All these data implied that miR-431 can directly bind to NRXN3 to regulate its expression.

### In vitro studies showed that miR-431 could promote glioma proliferation, migration and invasion by regulating NRXN3

**Fig. 6:**
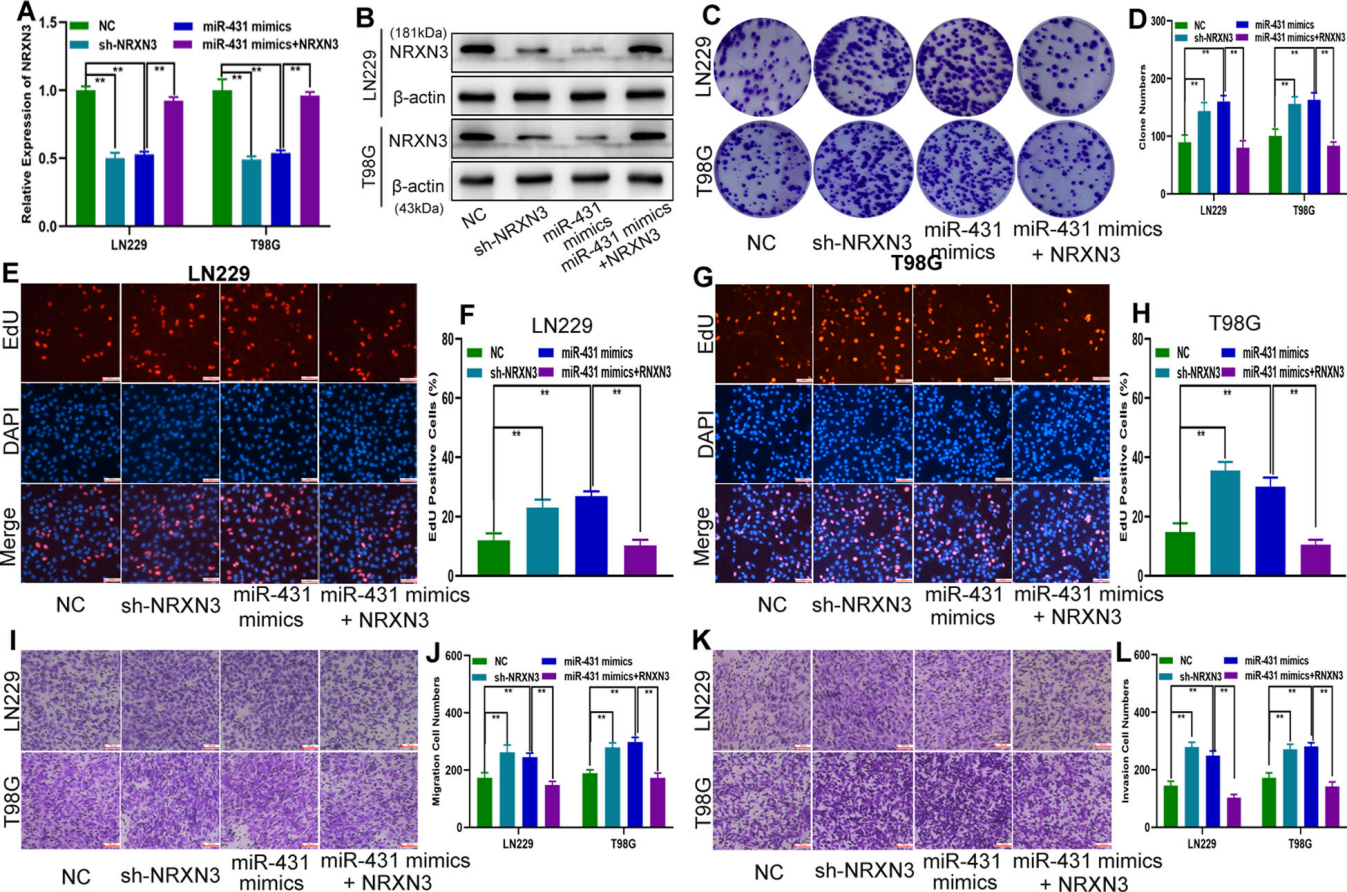
In vitro studies showed that miR-431 could promote glioma proliferation, migration and invasion by regulating NRXN3. **A**, **B** qRT-PCR and western blot analysis of NRXN3 expression in LN229 and T98G cell lines transfected with sh-NRXN3, miR-431 mimics, or miR-431 mimics together with NRXN3 plasmid. **C**, **D** Clone formation assay to analyse the proliferation of LN229 and T98G cells transfected with sh-NRXN3, miR-431 mimics or miR-431 mimics together with NRXN3 plasmid. **E**–**H** EdU assay to evaluate the proliferation of LN229 and T98G cells transfected with sh-NRXN3, miR-431 mimics or miR-431 mimics together with NRXN3 plasmid. **I**, **J** Transwell assay to analyse the migration of LN229 and T98G cells transfected with sh-NRXN3, miR-431 mimics or miR-431 mimics together with NRXN3 plasmid. **K**, **L** Transwell assay to detect invasion of LN229 and T98G cells transfected with sh-NRXN3, miR-431 mimics or miR-431 mimics together with NRXN3 plasmid. Each experiment was performed in triplicate. Data are expressed as mean ± SD, **P < 0.01.

To verify the mutual regulatory relationship between miR-431 and NRXN3, miR-431 mimics, shRNA targeting NRXN3 (sh-NRXN3) and miR-431 mimics together with an NRXN3 overexpression plasmid (miR-431 mimics + NRXN3) were chemically synthesized and transfected into LN229 and T98G cells (Fig. 6A, B). Functional assays indicated that miR-431 mimics and sh-NRXN3 promoted the proliferation, migration and invasion of glioma cells and that the promoting effect of miR-431 mimics could be restored by NRXN3 overexpression (Fig. 6C–L). Overall, these findings suggested that miR-431 can regulate the proliferation, migration and invasion of glioma cells by targeting NRXN3.

### NRXN3 inhibited glioma growth in vivo

**Fig. 7:**
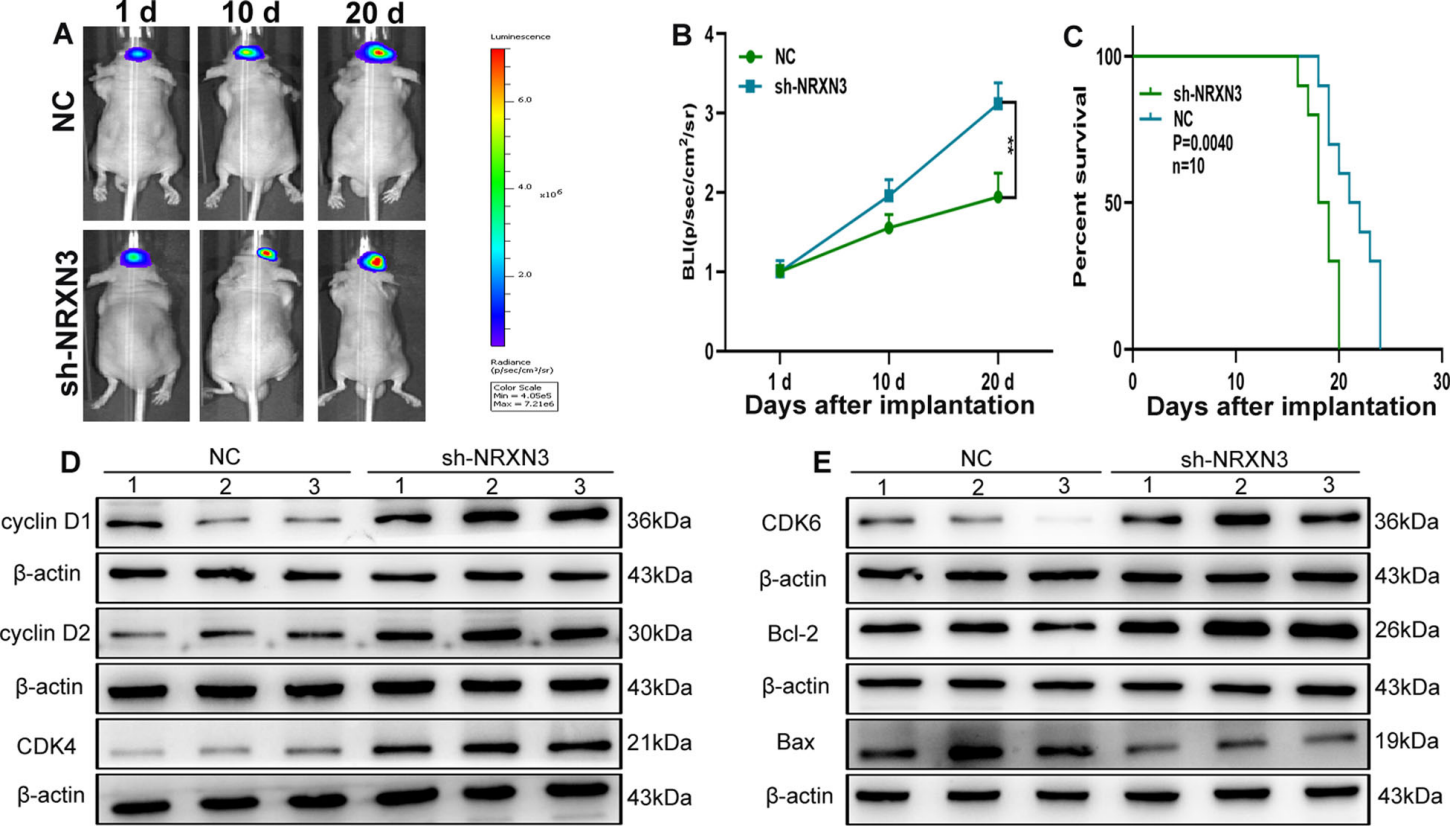
NRXN3 inhibits glioma growth in vivo. **A**, **B** Bioluminescent images of the intracerebral tumours of nude mice (10 mice in per group) were acquired on days 1, 10 and 20 after glioma cell implantation. **C** Overall survival was compared between the sh-NRXN3 and shNC groups with Kaplan–Meier survival analysis. **D**, **E** The expression of proliferation- and apoptosis-related proteins was detected by western blot analysis. Data are expressed as mean ± SD, **P < 0.01.

LN229 cells transfected with sh-NRXN3 or the corresponding negative control were applied to establish the orthotopic tumour model with stereotaxic intracerebral inoculation, which showed that silencing NRXN3 led to promotion of glioma growth in vivo and resulted in shorter survival of tumour-bearing mice (Fig. 7A–C). Western blot analysis and IHC showed that cyclin D1, cyclin D2, CDK4, CDK6, Ki-67, Bcl-2 and Bax were involved in the NRXN3-induced inhibitory effect on glioma growth (Figs. 7D, E and S6A). Additionally, TUNEL staining showed that TUNEL-positive cells decreased in the sh-NRXN3 group (Fig. S6B).

### Circ_0001367 inhibited the glioma malignancy phenotype by targeting the miR-431/NRXN3 axis

**Fig. 8:**
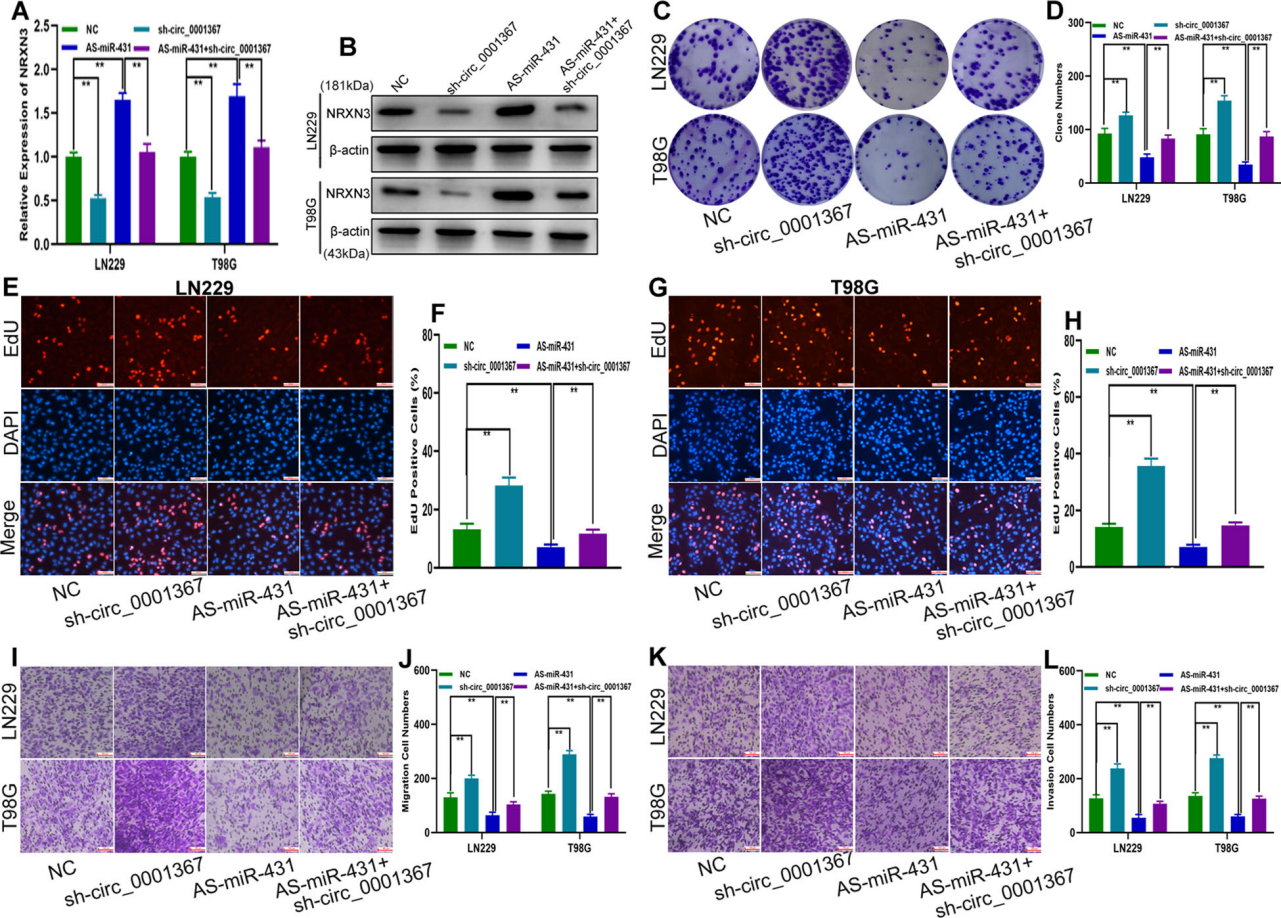
Circ_0001367 inhibited the glioma malignancy phenotype by targeting the miR-431/NRXN3 axis. **A, B** qRT-PCR and western blot analysis of NRXN3 expression in LN229 and T98G cell lines transfected with sh-circ_0001367, miR-431 inhibitors or sh-circ_0001367 + miR-431 inhibitors. **C**, **D** Clone formation assay to evaluate the proliferation of LN229 and T98G cells transfected with sh-circ_0001367, miR-431 inhibitors or sh-circ_0001367 + miR-431 inhibitors. **E**–**H** EdU assay to measure the proliferation of LN229 and T98G cells transfected with sh-circ_0001367, miR-431 inhibitors or sh-circ_0001367 + miR-431 inhibitors. **I**, **J** Transwell assay to detect the migration of LN229 and T98G cells transfected with sh-circ_0001367, miR-431 inhibitors or sh-circ_0001367 + miR-431 inhibitors. **K**, **L** Transwell assay to analyse the invasion of LN229 and T98G cells transfected with sh-circ_0001367, miR-431 inhibitors or sh-circ_0001367 + miR-431 inhibitors. Each experiment was performed in triplicate. Data are expressed as mean ± SD, **P < 0.01.

The preliminary data showed that circ_0001367 could sponge miR-431 to regulate NRXN3 expression. To further verify the integrity of the ceRNA pathway, rescue experiments were performed. qRT-PCR and western blot analysis demonstrated that knocking down circ_0001367 decreased NRXN3 expression, whereas miR-431 inhibitors increased NRXN3 expression at both the mRNA and protein levels, and the inhibitory effect of sh-circ_0001367 was restored by miR-431 inhibitors (Fig. 8A, B). Subsequent functional assays showed that sh-circ_0001367 promoted the proliferation, migration and invasion of glioma cells, whereas miR-431 inhibitors suppressed these biological behaviours of glioma cells. Simultaneously, the promoting effect of sh-circ_0001367 on the proliferation, migration and invasion of glioma cells could be partially restored by miR-431 inhibitors (Fig. 8C–L). These results demonstrated that circ_0001367 inhibited glioma progression by acting as a sponge of miR-431 to regulate NRXN3.

## Discussion

CircRNAs are an important class of ncRNAs that were thought to have no regulatory effects on biological activities when first discovered decades ago^25^. With the advancement of sequencing technology, biologists have accumulated a large number of RNA sequencing data sets and found that circRNAs are more commonly involved in gene regulation in human cells than linear RNAs^26^. Mounting studies have shown that circRNAs are widely expressed in various species and participate in the transcription and expression of genes, thus affecting biological physiological and pathological processes^27^. Mounting evidence has shown that circRNAs are significantly differentially expressed in tumours and closely related to tumour progression^28,29^. In the current study, according to circRNA expression profile and qRT-PCR, circ_0001367 was found to be downregulated in glioma tissues and associated with glioma patient survival, which suggested that circ_0001367 may function as a novel biomarker and potential target in the diagnosis and treatment of gliomas. Circ_0001367 is located on chromosome 3, and its role in human disease has never been studied thoroughly. Herein, in vitro assays indicated that circ_0001367 played a negative role in the proliferation, migration and invasion of glioma cells. Simultaneously, an in vivo assay also demonstrated that circ_0001367 could inhibit orthotopic xenograft growth. These results indicated that circ_0001367 played an important suppressive role in glioma growth and development, therefore the underlying mechanism merited clarification.

Recent studies suggest that circRNAs function in four common ways: (1) acting as miRNA sponges; (2) regulating RNA-binding proteins; (3) regulating gene transcription; and (4) being translated by ribosomes to polypeptides^30^. For circRNAs containing miRNA response elements, a large number of studies have reported that the adsorption of miRNAs is the most critical way for circRNAs to exert their biological functions, which can further regulate downstream target genes to influence malignant tumour progression, recurrence and chemoresistance. For example, circNTRK2 regulated oesophageal squamous cell carcinoma progression by targeting miR-140-3p^31^, circSHKBP1 promoted the progression of gastric cancer by sponging miR-582-3p^32^, and circ_0091570 inhibited hepatocellular cancer progression by sponging miR-1307^33^. MiR-431 is known to participate in the carcinogenesis of various cancers, such as pancreatic and neuroendocrine tumours^34^, breast cancer^35^ and colon cancer^36^. There was also a study which documented that miR-431 downregulation suppressed the viability of glioblastoma cells; however, the role of miR-431 and the related regulatory network has not been fully elucidated and still needs further investigation^37^. In the current study, miR-431 was found to be upregulated in both glioma cell lines and surgical glioma specimens. In addition, luciferase reporter and RIP assays revealed that miR-431 can be adsorbed by circ_0001367.

NRXN3, a member of the neurexin gene family, is involved in neuropsychiatric disorders and cancer progression^38–40^. NRXN3 participates in renal cell carcinoma cell adhesion^41^, breast cancer progression^42^ and glioma progression^43^. The current study found that NRXN3 was downregulated in gliomas and inhibited the proliferation, migration and invasion of glioma cells. A series of assays on molecular pathways verified that NRXN3 acted as a functional target of miR-431. In addition, rescue experiments indicated that circ_0001367 could regulate the expression and function of NRXN3 by sponging miR-431.

In conclusion, the current study demonstrated that circ_0001367 was downregulated in gliomas and could inhibit glioma progression by sponging miR-431 to regulate NRXN3 (Fig. S7). These results suggest that circ_0001367 may be a potential diagnostic biomarker and a therapeutic target against gliomas.

## Supplementary information

Figure S1

Figure S2

Figure S3

Figure S4

Figure S5

Figure S6

Figure S7

Table S1

Table S2

Table S3

Table S4

Table S5

Supplementary material files
