# Supplementary figures and images for: Circ_0001367 inhibits glioma proliferation, migration and invasion by sponging miR-431 and thus regulating NRXN3
Source: Cell Death Dis. 2021 May 25;12(6):536. doi: 10.1038/s41419-021-03834-1 (PMC8149867; doi:10.1038/s41419-021-03834-1)

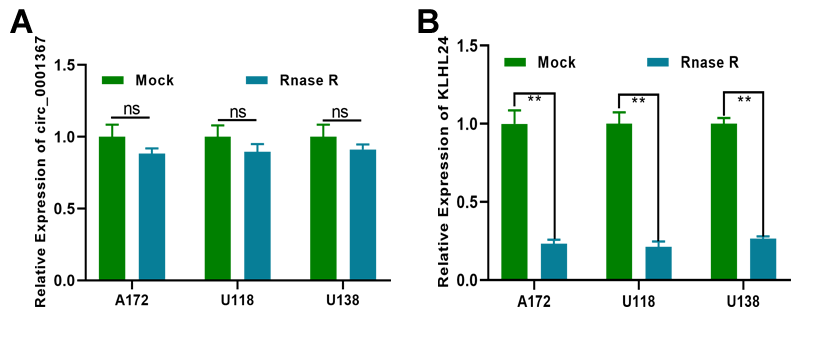

Supplement: Supplementary file 1 — Figure S1 [file 41419_2021_3834_MOESM1_ESM.tif]

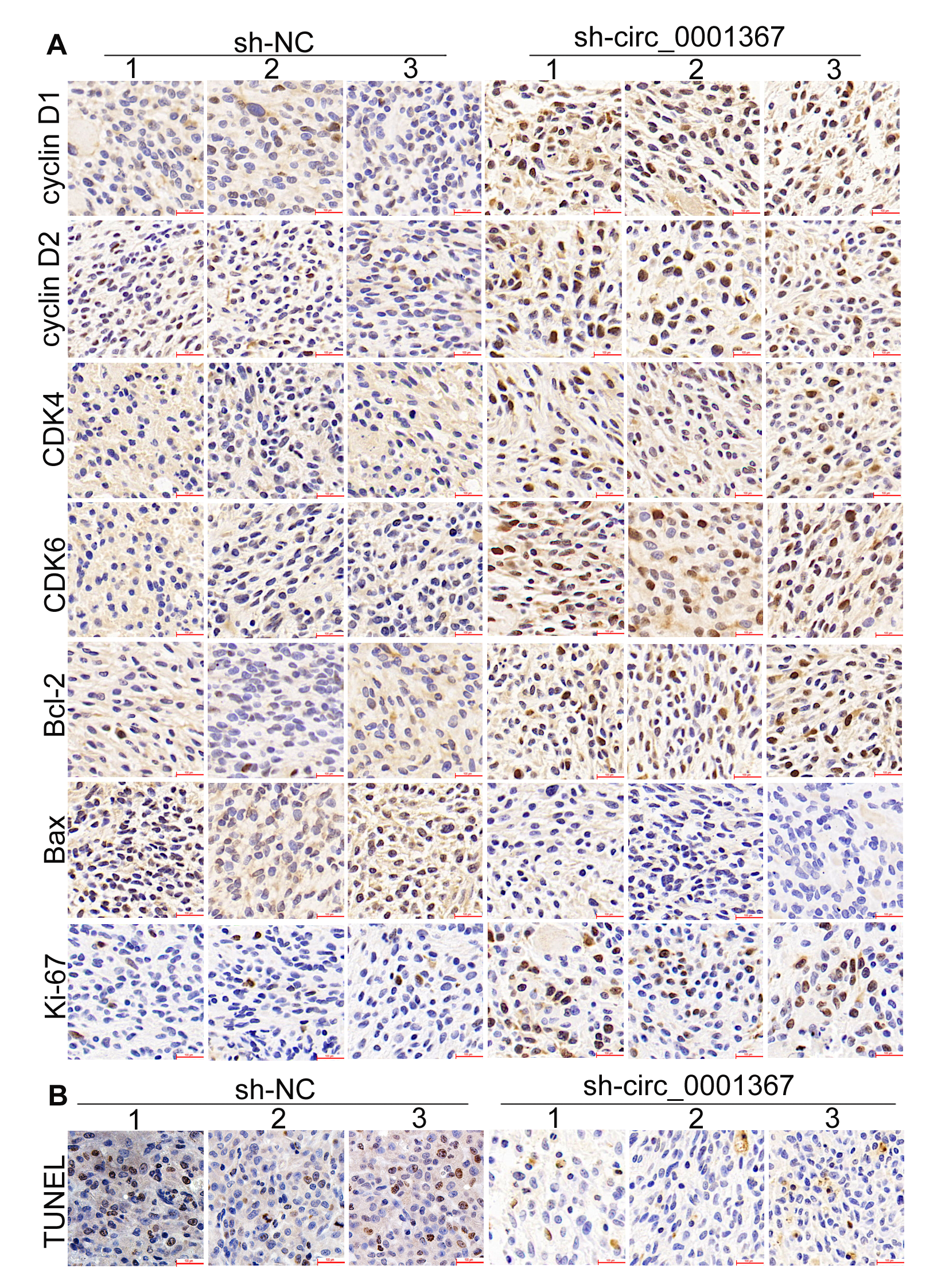

Supplement: Supplementary file 2 — Figure S2 [file 41419_2021_3834_MOESM2_ESM.tif]

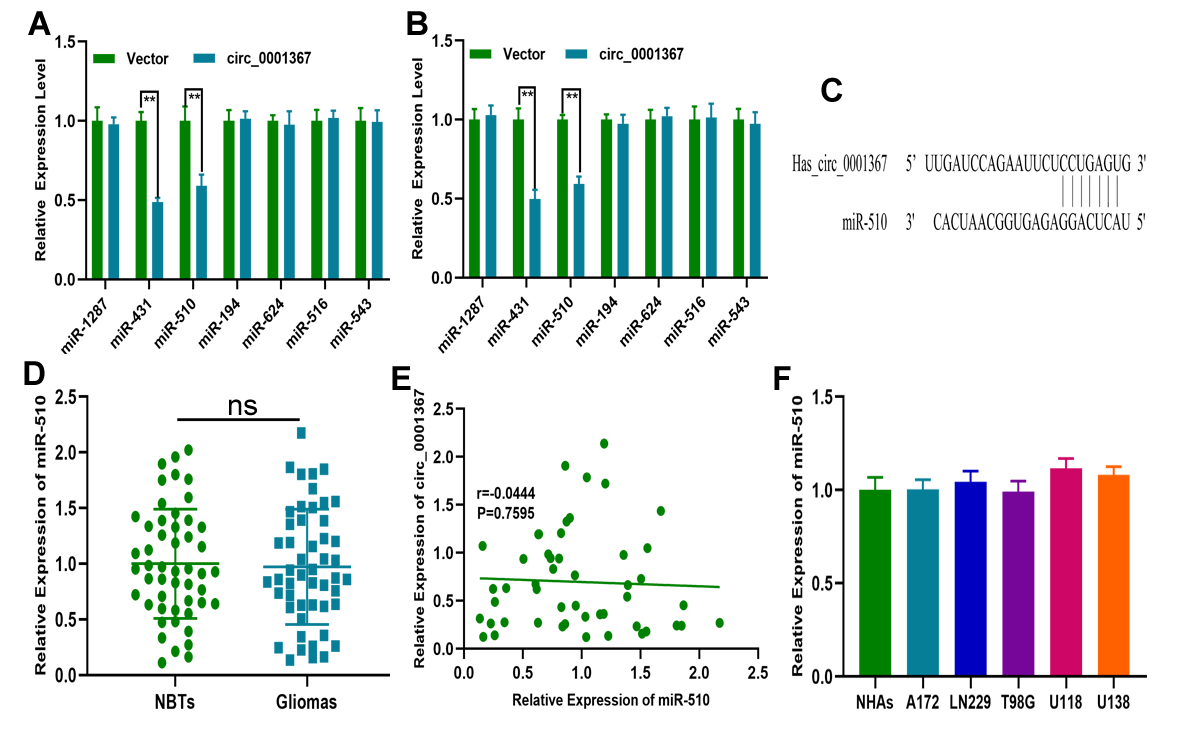

Supplement: Supplementary file 3 — Figure S3 [file 41419_2021_3834_MOESM3_ESM.tif]

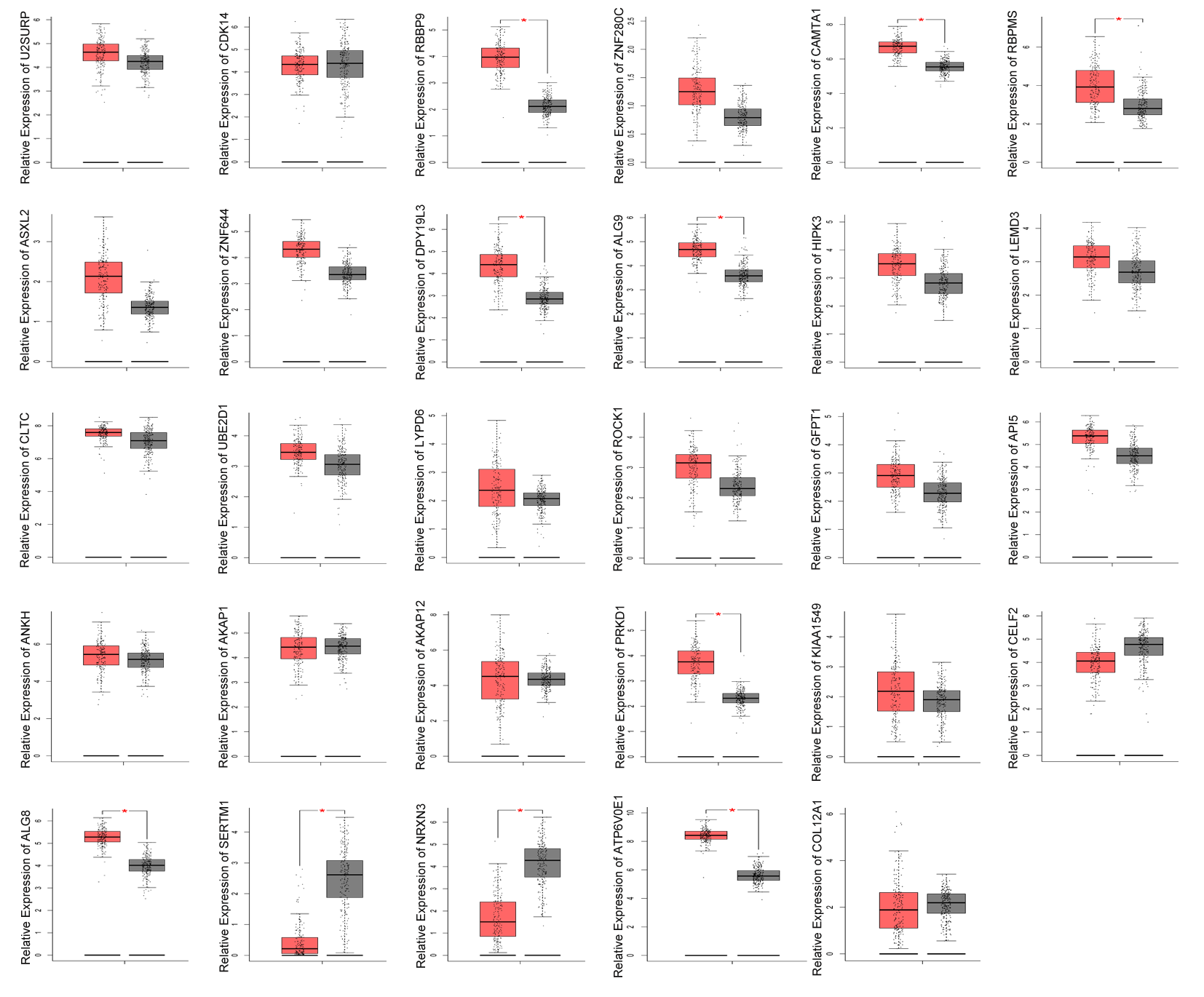

Supplement: Supplementary file 4 — Figure S4 [file 41419_2021_3834_MOESM4_ESM.tif]

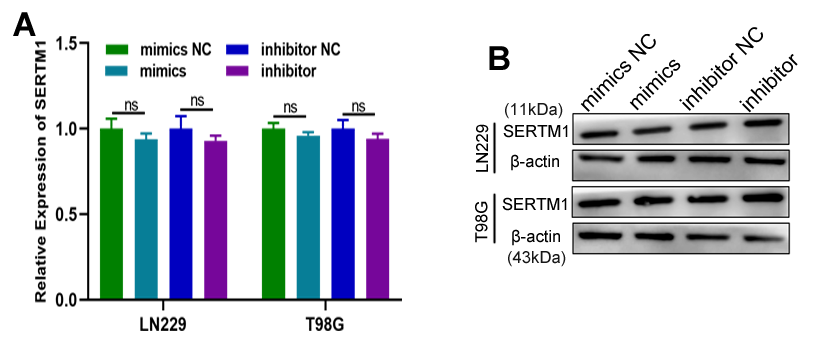

Supplement: Supplementary file 5 — Figure S5 [file 41419_2021_3834_MOESM5_ESM.tif]

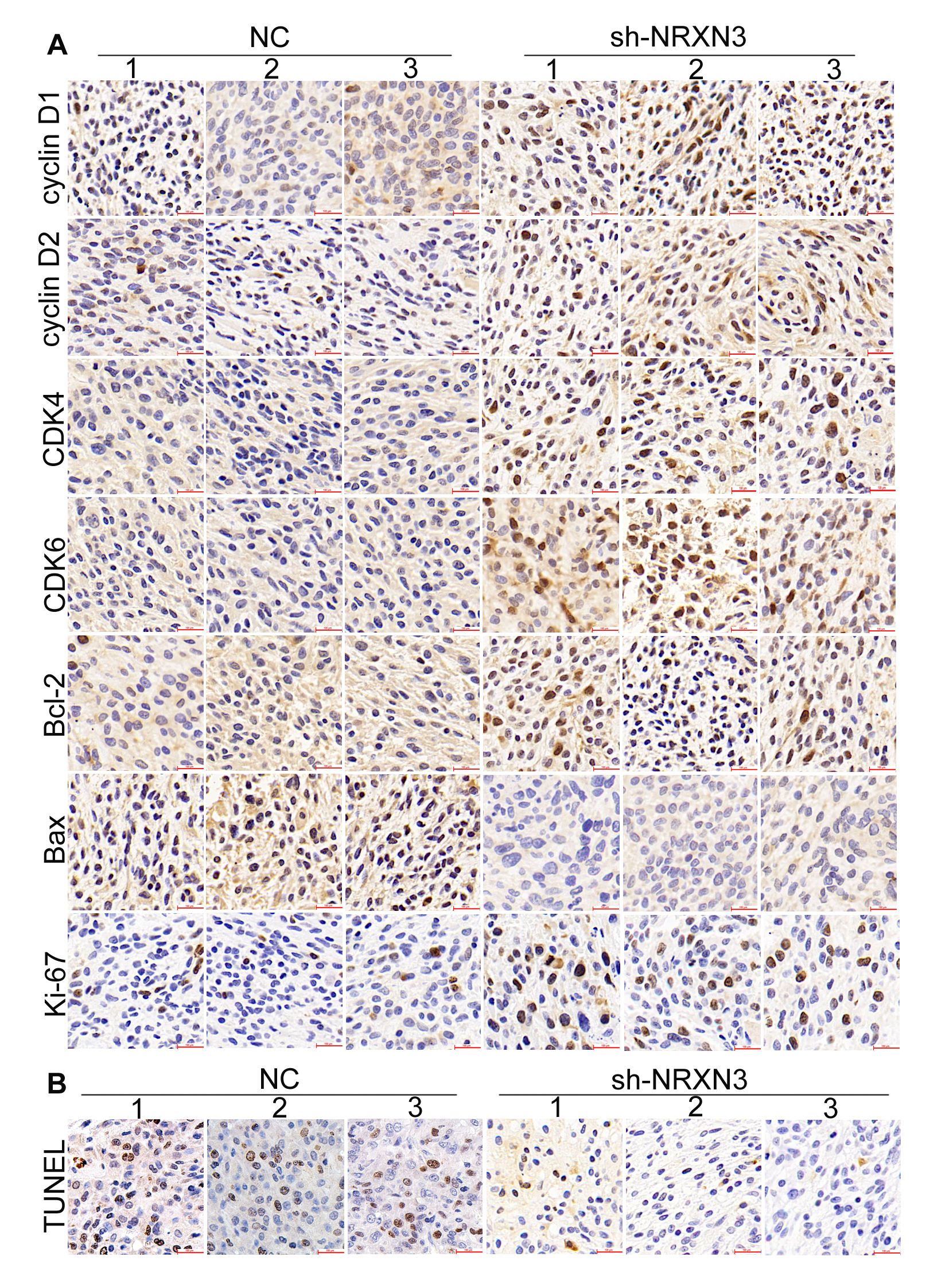

Supplement: Supplementary file 6 — Figure S6 [file 41419_2021_3834_MOESM6_ESM.tif]

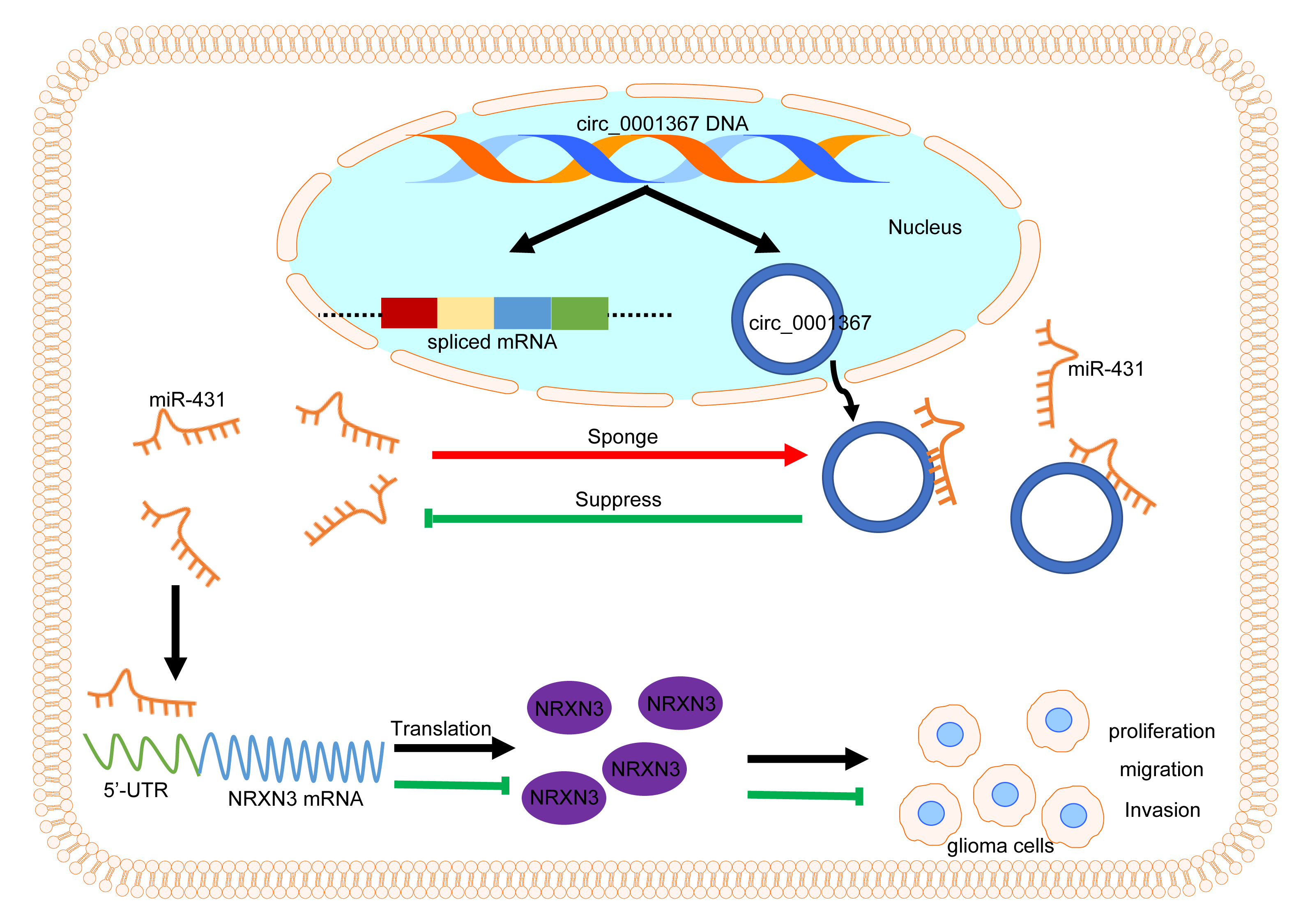

Supplement: Supplementary file 7 — Figure S7 [file 41419_2021_3834_MOESM7_ESM.tif]
