# Supplementary material for: Circ_0001367 inhibits glioma proliferation, migration and invasion by sponging miR-431 and thus regulating NRXN3
Source: Cell Death Dis. 2021 May 25;12(6):536. doi: 10.1038/s41419-021-03834-1 (PMC8149867; doi:10.1038/s41419-021-03834-1)
Supplement: Supplementary file 8 — Table S1 [file 41419_2021_3834_MOESM8_ESM.docx]

**Table. S1 The oligonucleotide sequences used for cell transfection assays.**

| **Names** | Sense (5’-3’) | Antisense (5’-3’) |
| --- | --- | --- |
| sh-circ_0001367-1 | GCCACGCAGGTGTGGAAATTA |  |
| sh-circ_0001367-2 | CTAGGCCACGCAGGTGTGGAA |  |
| sh-NC | UUCUCCGAACGUGUCACGUTTUGC |  |
| miR-431 mimics | UGUCUUGCAGGCCGUCAUGCA | CAUGACGGCCUGCAAGACAUU |
| miR-431 mimics NC | GCGUUAAUCUUUCGCGUUAUA | UAACGCGAAAGAUUAACGCUU |
| miR-431 inhibitors | UGCAUGACGGCCUGCAAGACA |  |
| miR-431 inhibitors NC | GGCAAACAUUAACGCAAGUAU |  |
| sh-NRXN3 | GCAAAGGACCAGAGACCTTGT |  |
| sh-NRXN3 NC | TTCTCCGAACGTGTCACGTCT |  |
