# Supplementary material for: Circ_0001367 inhibits glioma proliferation, migration and invasion by sponging miR-431 and thus regulating NRXN3
Source: Cell Death Dis. 2021 May 25;12(6):536. doi: 10.1038/s41419-021-03834-1 (PMC8149867; doi:10.1038/s41419-021-03834-1)
Supplement: Supplementary file 9 — Table S2 [file 41419_2021_3834_MOESM9_ESM.docx]

**Table. S2. The primers used in this study.**

| Gene | Forward (5’-3’) | Reverse (5’-3’) |
| --- | --- | --- |
| circ_0001367 | TGGGTCTATCGTGCCGTTGA | GGACATCATTTCATTCCCAAGTA |
| miR-431-5p | ACGCGTGTCTTGCAGGCCGT | ATCCAGTGCAGGGTCCGAGG |
| miR-510-5p | TAGCTCAGGAGAGTGGCAATCA | GTGCAGGGTCCGAGGT |
| NRXN3 | GCTGAGAACAACCCCAATA | ATGCTGGCTGTAGAGCGATT |
| β-actin | CGTGACATTAAGGAGAAGCTG | CTAGAAGCATTTGCGGTGGAC |
| U6 | CTCGCTTCGGCAGCACA | AACGCTTCACGAATTTGCGT |
