# Supplementary material for: Circ_0001367 inhibits glioma proliferation, migration and invasion by sponging miR-431 and thus regulating NRXN3
Source: Cell Death Dis. 2021 May 25;12(6):536. doi: 10.1038/s41419-021-03834-1 (PMC8149867; doi:10.1038/s41419-021-03834-1)
Supplement: Supplementary file 10 — Table S3 [file 41419_2021_3834_MOESM10_ESM.docx]

**Table. S3. The primary antibody used in Western blot analysis.**

| Names | Molecular Weight | Catalog # | Application | Company |
| --- | --- | --- | --- | --- |
| Cyclin D1 | 36kDa | MAB14432 | WB/IHC | Abnova |
| Cyclin D2 | 30kDa | MAB1852 | WB/IHC | Abnova |
| CDK4 | 21kDa | PAB12995 | WB/IHC | Abnova |
| CDK6 | 36kDa | 14052-1-AP | WB/IHC | Proteintech |
| Bcl-2 | 26kDa | 12789-1-AP | WB/IHC | Proteintech |
| Bax | 19kDa | 33-6400 | WB/IHC | Invitrogen |
| NRXN3 | 181kDa | Ab230635 | WB/IH | Abcam |
| β-actin | 43kDa | 14395-1-AP | WB | Proteintech |
