# Supplementary material for: Circ_0001367 inhibits glioma proliferation, migration and invasion by sponging miR-431 and thus regulating NRXN3
Source: Cell Death Dis. 2021 May 25;12(6):536. doi: 10.1038/s41419-021-03834-1 (PMC8149867; doi:10.1038/s41419-021-03834-1)
Supplement: Supplementary file 11 — Table S4 [file 41419_2021_3834_MOESM11_ESM.docx]

**Table S4. Nine downregulated circRNAs in glioma were identified from circRNA expression profiles.**

| **Circ ID** | **Location** | **Gene Symbol** | **Fold change** |
| --- | --- | --- | --- |
| Hsa_circ_0015663 | Chr10 (7217951-7244484) | SFMBT2 | 0.170928816 |
| Hsa_circ_0008225 | Chr10 (225933-267296) | ZMYND11 | 0.186405522 |
| Hsa_circ_0026260 | Chr6 (74189454-74192343) | MTO1 | 0.189070896 |
| Hsa_circ_0001367 | Chr3 (183361267-183369064) | KLHL24 | 0.211689347 |
| Hsa_circ_0064555 | Chr3 (18419661-18462483) | SATB1 | 0.235738913 |
| Hsa_circ_0030746 | Chr4 (144449020-144451679) | SMARCA5 | 0.247383147 |
| Hsa_circ_0017127 | Chr16 (68308593-68309152) | SLC7A6 | 0.275247693 |
| Hsa_circ_0006467 | Chr20 (33057852-33069011) | ITCH | 0.291049958 |
| Hsa_circ_0010290 | Chr17 (57725600-57758839) | CLTC | 0.295822554 |
