# Supplementary material for: Circ_0001367 inhibits glioma proliferation, migration and invasion by sponging miR-431 and thus regulating NRXN3
Source: Cell Death Dis. 2021 May 25;12(6):536. doi: 10.1038/s41419-021-03834-1 (PMC8149867; doi:10.1038/s41419-021-03834-1)
Supplement: Supplementary file 12 — Table S5 [file 41419_2021_3834_MOESM12_ESM.docx]

**Table. S5 The list of 29 mRNAs which may be biological targets of miR-431**

| **Candidate mRNAs** | **Full name** |
| --- | --- |
| U2SURP | U2 snRNP associated SURP domain containing |
| CDK14 | Cyclin dependent kinase 14 |
| RBBP9 | RB binding protein 9, serine hydrolase |
| ZNF280C | Zinc finger protein 280C |
| CAMTA1 | Calmodulin binding transcription activator 1 |
| RBPMS | RNA binding protein, mRNA processing factor |
| ASXL2 | Additional sex combs like 2, transcriptional regulator |
| ZNF644 | Zinc finger protein 644 |
| DPY19L3 | Dpy-19 like C-mannosyltransferase 3 |
| ALG9 | alpha-1,2-mannosyltransferase |
| HIPK3 | Homeodomain interacting protein kinase 3 |
| LEMD3 | LEM domain containing 3 |
| CLTC | Clathrin heavy chain |
| UBE2D1 | Ubiquitin conjugating enzyme E2 D1 |
| LYPD6 | LY6/PLAUR domain containing 6 |
| ROCK1 | Rho associated coiled - coil containing protein kinase 1 |
| GFPT1 | Glutamine--fructose-6-phosphate transaminase 1 |
| API5 | Apoptosis inhibitor 5 |
| ANKH | ANKH inorganic pyrophosphate transport regulator |
| AKAP1 | A-kinase anchoring protein 1 |
| AKAP12 | A-kinase anchoring protein 12 |
| PRKD1 | Protein kinase D1 |
| KIAA1549 | KIAA1549 |
| CELF2 | CUGBP Elav-like family member 2 |
| ALG8 | alpha-1,3-glucosyltransferase |
| SERTM1 | Serine rich and transmembrane domain containing 1 |
| NRXN3 | Neurexin 3 |
| ATP6V0E1 | ATPase H^+^ transporting V0 subunit e1 |
| COL12A1 | Collagen type XII alpha 1 chain |
