# Supplementary material for: Circ_0001367 inhibits glioma proliferation, migration and invasion by sponging miR-431 and thus regulating NRXN3
Source: Cell Death Dis. 2021 May 25;12(6):536. doi: 10.1038/s41419-021-03834-1 (PMC8149867; doi:10.1038/s41419-021-03834-1)
Supplement: Supplementary file 13 — Supplementary material files [file 41419_2021_3834_MOESM13_ESM.docx]

**Additional files**

**Supplementary tables and figures**

**Table S1. The oligonucleotide sequences used for the cell transfection assay.**

**Table S2. The primers used in this study.**

**Table S3. Primary antibodies used in Western blot assay.**

**Table S4. Nine downregulated circRNAs in glioma were identified from circRNA expression profiles.**

**Table S5. List of 29 mRNAs that may be biological targets of miR-431.**

**Figure. S1. Resistance of circ_0001367 RNA to RNase R in A172, U118 and U138 cells.**

**Figure S2. The related indicators were detected in sh-circ_0001367 and sh-NC groups.**

**(A)** The expression of proliferation- and apoptosis-related proteins was analyzed with immunohistochemistry (IHC).

**(B)** TUNEL staining of the sh-circ_0001367 and sh-NC groups.

**Figure. S3. Relative expression of miR-510 in glioma clinical specimens and cell lines.**

**(A-B)** Relative expression of candidate miRNAs in LN229 and T98G cells transfected with the circ_0001367 overexpression plasmid.

**(C)** The putative binding sites of miR-510 on circ_0001367.

**(D)** The expression of miR-510 in glioma clinical specimens and matching adjacent normal brain tissues (NBTs) was examined by qRT-PCR.

**(E)** Pearson correlation analysis was performed on the relationship between circ_0001367 and miR-510.

**(F)** The expression of miR-510 in glioma cells.

**Figure. S4. The expression of 29 candidate mRNAs in gliomas from the public database GEPIA.**

**Figure. S5. qRT-PCR and Western blot analysis indicated that there was no close relationship between SERTM1 and miR-431.**

**Figure S6. The related indicators were detected in sh-NRXN3 and NC groups.**

**(A)** The expression of proliferation- and apoptosis-related proteins was analyzed with immunohistochemistry.

**(B)** TUNEL staining of the sh-NRXN3 and NC groups.

**Figure S7. Schematic diagram of the circ_0001367-mediated pathway in glioma**.
